# Responses of genes involved in cell cycle control to diverse DNA damaging chemicals in human lung adenocarcinoma A549 cells

**DOI:** 10.1186/1475-2867-5-28

**Published:** 2005-08-24

**Authors:** Huijun Zhu, Catherine Smith, Charles Ansah, Nigel J Gooderham

**Affiliations:** 1 Molecular Toxicology (Biological Chemistry), Division of Biomedical Sciences, Faculty of Life Sciences, Imperial College London, Sir Alexander Fleming Building London, SW7 2AZ, UK

## Abstract

**Background:**

Many anticancer agents and carcinogens are DNA damaging chemicals and exposure to such chemicals results in the deregulation of cell cycle progression. The molecular mechanisms of DNA damage-induced cell cycle alteration are not well understood. We have studied the effects of etoposide (an anticancer agent), cryptolepine (CLP, a cytotoxic alkaloid), benzo [a]pyrene (BaP, a carcinogenic polycyclic aromatic hydrocarbon) and 2-amino-1-methyl-6-phenylimidazo [4,5-b]pyridine (PhIP, a cooked-meat derived carcinogen) on the expression of cell cycle regulatory genes to understand the molecular mechanisms of the cell cycle disturbance.

**Results:**

A549 cells were treated with DMSO or chemicals for up to 72 h and periodically sampled for cell cycle analysis, mRNA and protein expression. DMSO treated cells showed a dominant G1 peak in cell cycle at all times examined. Etoposide and CLP both induced G2/M phase arrest yet the former altered the expression of genes functioning at multiple phases, whilst the latter was more effective in inhibiting the expression of genes in G2-M transition. Both etoposide and CLP induced an accumulation of p53 protein and upregulation of p53 transcriptional target genes. Neither BaP nor PhIP had substantial phase-specific cell cycle effect, however, they induced distinctive changes in gene expression. BaP upregulated the expression of CYP1B1 at 6–24 h and downregulated many cell cycle regulatory genes at 48–72 h. By contrast, PhIP increased the expression of many cell cycle regulatory genes. Changes in the expression of key mRNAs were confirmed at protein level.

**Conclusion:**

Our experiments show that DNA damaging agents with different mechanisms of action induced distinctive changes in the expression pattern of a panel of cell cycle regulatory genes. We suggest that examining the genomic response to chemical exposure provides an exceptional opportunity to understand the molecular mechanism involved in cellular response to toxicants.

## Background

Many chemical carcinogens and therapeutic agents interact with cells, leading to temporary/permanent cell growth arrest, genetic modification or cell death. The ultimate effect of a chemical on cells is largely determined by the chemical's ability to elicit genomic response. The recent launch of the National Institutes of Health NCI Chemical Genomics Initiative [1] heralds a new era of chemical-genome research. In the current study, we have used chemical-genomics and phenotypic expression to understand the molecular mechanisms involved in cellular response to chemical exposure.

We have examined four chemicals. Etoposide, a topoisomerase II inhibitor, induces DNA double strand breaks by promoting the formation of cleavable DNA-protein complexes and causes cell cycle arrest at S phase or G2/M phase dependent on the cell type [2,3]. Cryptolepine (CLP), an alkaloid extracted from the West African climbing shrub *Cryptolepis sanguinolenta*, interferes with topoisomerase II and inhibits DNA synthesis and is potently cytotoxic to tumor cells [4-6]. Benzo(a)pyrene (BaP), one of the polycyclic aromatic hydrocarbons (PAHs) derived from incomplete combustion of organic matter, is an archetypal procarcinogen. Epidemiological studies indicate a positive link between exposure to BaP and the occurrence of human cancers [7-9]. BaP exerts its genotoxicity *via *cytochrome P450-mediated metabolism, namely CYP1A1 and CYP1B1, to form electrophiles that covalently bind to DNA [10,11]. The expression of *CYP1A1 *and *CYP1B1 *can be induced by the activation of aryl hydrocarbon receptor (AhR), which is a ligand-activated transcription factor [12]. Upon binding to its ligands, such as dioxin and PAHs, AhR translocates to the nucleuswhere it complexes with ARNT to stimulate the transcription of genes via transactivation through enhancer domains known as AHR-, dioxin-, or xenobiotic-response elements [13,14]. The cytochrome P450 *Cyp1 *family, including *CYP1B1 *and *CYP1A1*, as well as several phase II detoxification genes are among those regulated by AhR [15,16]. Studies also provide evidence that AHR participates in the modulation of the transcriptional program at least in part by associating with additional transcription factors [17,18]. Such associations may be responsible for the effects of the ligand-activated AHR in the regulation of proliferation [19]. 2-Amino-1-methyl-6-phenylimidazo [4,5-b]pyridine (PhIP), a diet-derived heterocyclic amine formed during the cooking of meat [20], is a rodent carcinogen and suspected human carcinogen. It is known that PhIP and other heterocyclic amines are metabolized chiefly by CYP1A2 but also CYP1A1 and CYP1B1 to form electrophiles that bind to DNA to form DNA adducts [21-23].

In the current study we have used A549 human lung adenocarcinoma cells to examine the cellular and genomic responses to the chosen DNA reactive chemicals. The alveolar epithelial type II cell-derived A549 cells have been extensively used to test the cytotoxicity of therapeutic agents and environment toxicants [24]. These cells express aryl hydrocarbon receptor (AhR) [25], providing a useful model for studying AhR mediated gene regulation. Cell cycle analysis combined with cDNA Microarray assay allowed us to distinguish the molecular mechanisms of the cell cycle disturbance induced by the different chemicals and to more precisely predict the fate of cells after chemical exposure.

## Results

### Effects of treatments on cell cycle

**Figure 1:**
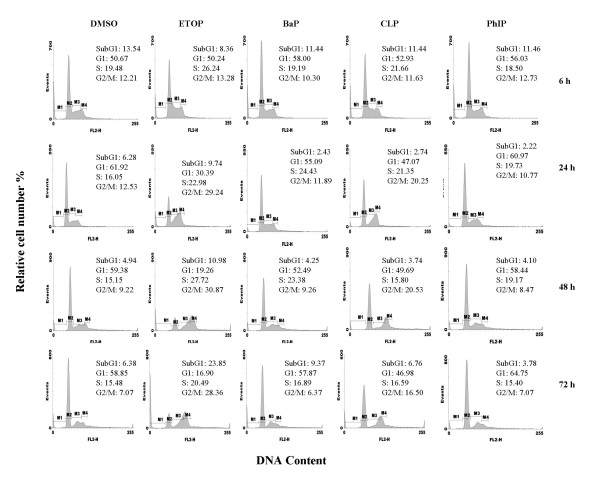
Effects of chemical treatment on cell cycle. A549 cells were treated with DMSO (< 0.1% v/v), etoposide (10 μM), BaP (25 μM), CLP (2.5 μM) or PhIP (50 μM) for the times indicated. Cell cycle distribution was assessed by flow cytometry. The gating represents % cells in each phase of the cell cycle. M1: sub-G1; M2:G0/G1 phase; M3: S phase; M4: G2/M phase.

Cell growth status was examined by microscopy and flow cytometry. Cells treated with DMSO, BaP and PhIP, but not etoposide and CLP, reached confluence after 24 h, as examined under microscope (data not shown). Figure 1 shows that in all DMSO treated samples, a predominant number of cells distributed in G1 phase of the cell cycle. In comparison, treatment with etoposide induced a time dependent decrease in the number of cells in G1 phase and an accumulation of cells in G2/M phase of the cell cycle. Cells treated with CLP displayed a cell cycle profile with elevated G2/M phase peak after 24 h. In comparison, BaP and PhIP had no persistent cell cycle specific effects. At 72 h, the sample treated with etoposide exhibited a nearly 2–3 fold increase in subG1 phase cells, compared with samples treated differently. Consistent with the occurrence of subG1 cells, we also observed more floating cells in the etoposide treated sample compared to the other treatments at 72 h, suggesting that etoposide induced cytoxicity at this late timepoint.

### Changes in the expression of P53 and its target genes in response to chemical treatments

**Figure 2:**
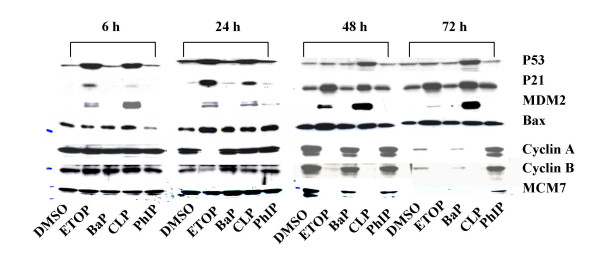
Western blots of p53, p53-transcriptional target gene proteins and proteins involved in cell cycle execution. Lysates from A549 cells treated as described in Fig. 1. were subjected to SDS-PAGE, electroblotted onto nitrocellulose and probed for specific immunoreactive proteins.

**Figure 3:**
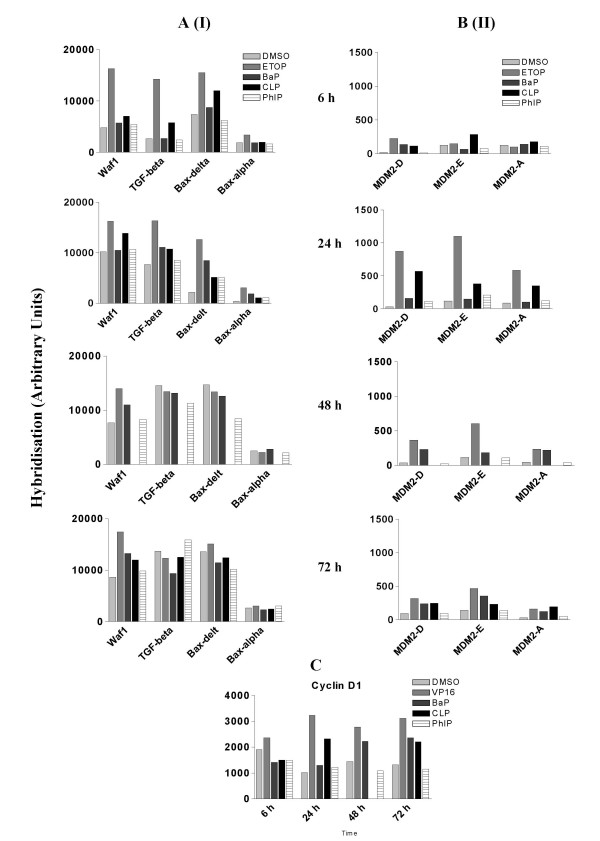
Effects of chemical treatment on mRNA levels of p53 target genes and cyclin D1 in A549 cells that were treated as described in Fig. 1 were measured using cDNA microarray hybridisation. A. p53 transcriptional targets (I). B. p53 transcriptional targets (II). C. Cyclin D1. GenBank accession number for each gene is displayed in table 1.

p53 is well-established as a primary responder to cellular and genetic stress [26]. Many of the p53 transcriptional target genes are involved in the cell cycle checkpoints. We have examined the levels of p53 protein together with some of its transcriptional target genes in response to different chemical treatments (table 1, group 1). Figure 2 shows a low level of p53 protein in DMSO treated cells. The expression levels of *WAF1 *and *TGF-β *mRNA, the protein products of which are involved in the G1/S transition cell cycle checkpoint [27,28], increased with time in DMSO treated cells (Fig. 3A). This pattern of change in the expression of *WAF1 *and *TGF-β *indicates that prolonged culture caused cell cycle arrest at G1 phase, which was supported by the observations that the number of cells in G1 phase was increased after 24 h compared with that at 6 h (Fig, 1). Indeed, after 24 h, cells treated with DMSO formed a uniform confluent monolayer. Exposure to etoposide and CLP induced an accumulation of p53 at 6 h and 24 h (Fig. 2). Consistent with this, *WAF1*, *TGF-β*, *BAX*, *MDM2 *all showed a positive response to both agents within 24 h at mRNA level (Fig. 3A, 3B). Although the ability for etoposide to induce the accumulation of p53 was largely diminished after 48 h (Fig. 2), exposure to the chemical was still effective in up-regulating *WAF1 *and *MDM2 *(Fig. 3A, 3B) Western blotting analysis also confirmed that exposure to etoposide and CLP upregulated the expression of proteins WAF1, MDM2 family members and BAX as early as 6 h (Fig. 2). Exposure to BaP and PhIP, on the other hand, increased the expression of *BAX *at 24 h (Figs. 2, 3A), but had little effects on any of the other p53 gene targets during the 72 h of treatment compared with the DMSO control (Figs. 2, 3A, 3B), consistent with the inability of these two chemicals to induce the accumulation of p53 protein (Fig. 2). These results suggest that exposure to both etoposide and CLP induced p53 activation, whilst etoposide, BaP and PhIP may also activate p53-independent pathways to regulate the expression of the so-called p53 target genes in A549 cells.

**Table 1:** Genes and their accession number in Genbank

**Genes**	**Access. No**	**Genes**	**Access. No**
**Group 1**		**Group 4**	
Waf	U03106	CDC25C	M34065
TGF-beta	AB000584	CENPf	U30872
Bax-delt	U19599	NEK2	Z2906
Bax-alpha	L22473	BUB1	AF053305
MDM2-D	U33202	BUB1B	AF053306
MMD2-A	U33199	TTK	M86699
MDM2-E	U33203		
			
**Group 2**		**Group 5**	
Cyclin D1	M64349	CDC34	L22005
CDK4	U37022	CDC45L	AJ223728
Cyclin E	M74093	MCM2	D21063
E2F	M96577	MCM6	D84557
DP1	L23959	MCM7	D55716
Rb	M15400	MCM8	D55083
			
**Group 3**		**Group 6**	
BIRC3	U75285	CYP11A	M14565
CCNB1	M25753	CYP1A1	X02612
CCNA2	X51688	CYP1B1	U03688
CDKN3	L25876	CYP24	L13286
HSCDC6	U77949	CYP2A7	M33317
MYT1	U56816	CYP2B6	M29874
		CYP4B1	J02871
		CYP51	U23942

Group 1: P53 target genes; group 2: genes involved in G1/S transition; group 3: genes involved in G2/M phase transition; group 4: genes involved in mitosis; group 5: genes involved in DNA replication initiation; group 6: CYP genes.

### Effects on genes involved in G1/S transition

Apart from the p53 target genes *WAF1 *and *TGF-β*, we have also examined the expression of other genes involved in the G1-S phase transition (genes in group 2 in table 1). These genes showed no appreciable response to the treatments (data not shown) with the exception of *CYCLIN D1*, which appeared to be upregulated by exposure to etoposide, CLP and BaP (Fig. 3C). The effects of all chemical treatments on the expression of G1/S transition genes seemed to be insufficient to cause cell cycle arrest in G1 phase.

### Effects on genes involved in G2/M transition

**Figure 4:**
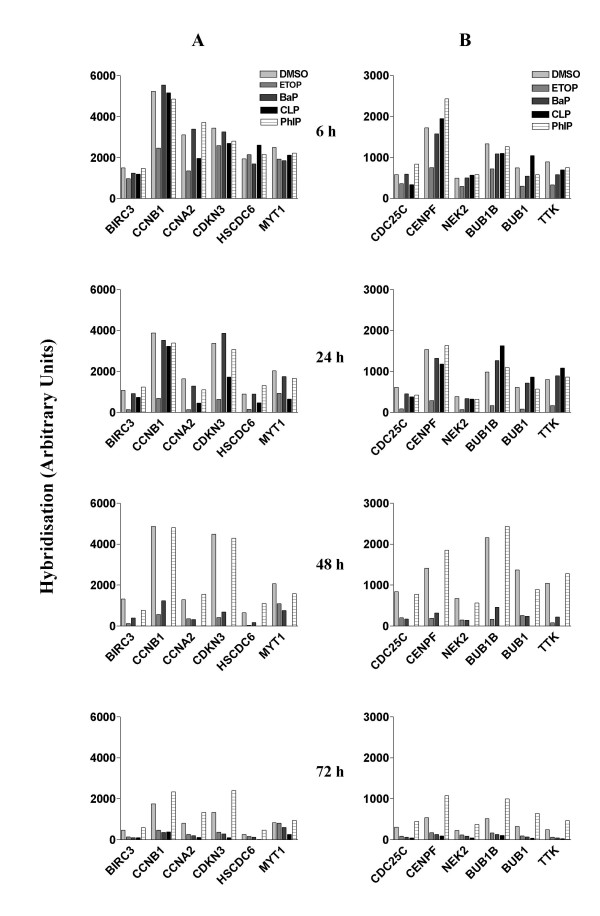
Effects of chemical treatment on mRNA levels of genes involved in G2/M transition and mitosis. mRNA levels in A549 cells that were treated as described in Fig. 1 were measured using cDNA microarray hybridisation. A. G2/M genes. B. Mitosis genes. GenBank accession number for each gene is displayed in table 1.

In DMSO treated cells, the expression levels of genes involved in G2/M phase transition (Table 1, group 3) were lowest at 72 h (Fig. 4A), suggesting a low growth potential in these cells at this stage. Etoposide persistently inhibited the expression of all these genes. The effects on *CYCLIN A (CCNA2) *and *CYCLIN B1 (CCNB1) *were observed as early as 6 h. CLP inhibited the expression of many of these genes within 24 h and all of them by 72 h. Interestingly, exposure to BaP also inhibited the expression of the majority of these genes, although the effects were not observed before 48 h. Exposure to PhIP, on the other hand, upregulated some of these genes at the later timepoints, though the effects were not substantial. These results suggest that etoposide is the most effective agent in inhibiting cell cycle progression through G2/M phase transition. The marginal effect elicited by PhIP indicated a small growth stimulus competing against confluence-related growth inhibition.

### Effects on genes involved in mitosis

The expression of genes involved in mitosis (Table 1, group 4) in cells treated with DMSO was lowest at 72 h, suggesting that the rate of cell division was lowest at this time point (Fig. 4B). Exposure to etoposide repressed the expression of all these genes within 6 h of treatment. In comparison, CLP showed no such inhibition up to 24 h of treatment, although inhibition was evident at 72 h. Exposure to BaP, again, had no effects on the expression of these genes before 24 h, but inhibited the expression of all these genes after 48 h of treatment. In complete contrast, cells treated with PhIP expressed higher levels of these mitosis-related genes at the 72 h time point. This suggests that PhIP either had the potential to delay the growth inhibition initiated by confluence or exerted a mild growth stimulation effect.

### Effects on genes involved in DNA replication initiation

**Figure 5:**
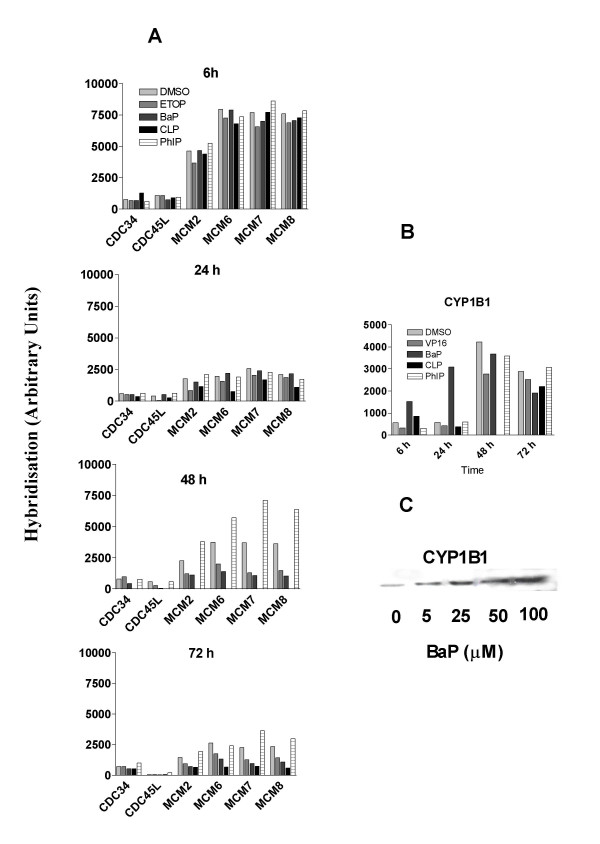
Effects of chemical treatment on expression of genes involved in DNA replication initiation and CYP1B1. mRNA levels in A549 cells that were treated as described in Fig. 1 were measured using cDNA microarray hybridization and protein levels by Western blotting. A. mRNA levels of DNA replication initiation genes. B. mRNA levels of CYP1B1 C. Expression of CYP1B1 protein. GenBank accession number for each gene is displayed in table 1.

In cells treated with DMSO, the expression of genes involved in DNA replication initiation (Table 1, group 5) was highest at 6 h (Fig. 5A). Although the expression of most of these genes had dramatically decreased at 24 h, there was no time-dependent further decrease thereafter. This pattern of change suggests that the process of DNA synthesis initiation was still active when cell growth had reached confluence. Exposure to etoposide, CLP and BaP all caused inhibitory effects on the expression of many of these genes, although the effects of BaP were only apparent after 48 h. Again, in contrast to the other treatments, PhIP appeared to upregulate the expression of many genes in this group at the 48 h and 72 h time points. These results suggest that cells treated with PhIP were more active in initiating DNA synthesis than cells treated with DMSO and with the other three agents, again supporting a growth stimulating effect.

Western blotting was also used to examine the expression level of a number of gene products (Fig. 2). Consistent with the data seen at the mRNA level, the protein levels of cyclin A and cyclin B were much lower at 72 h than any other time point in DMSO treated cells (Fig. 2). Exposure to etoposide and CLP decreased the levels of cyclin A and cyclin B, consistent with their effects on the mRNA levels of these proteins. Exposure to BaP, which reduced the mRNA levels of cyclin A and cyclin B at late time points, also reduced the levels of these proteins. Finally and again in contrast to the other treatments, at 72 h PhIP treatment induced the expression of cyclin A, cyclin B and MCM7 proteins (Fig. 2) consistent with a growth stimulatory effect. These results clearly show that changes at mRNA level resulted in changes at protein level. The temporal aspect of altered expression of mRNA to altered expression of protein depends upon the stability of the respective message and protein.

### Effect on CYP1B1

The expression level of *CYP1B1 *in DMSO treated cells was relatively low at 6 h and 24 h but was about 10-fold higher after 48 h (Fig. 5B). Compared with DMSO, exposure to BaP induced a time-dependent upregulation of *CYP1B1 *from 6 h to 24 h (Fig. 5B). The induction of CYP1B1 protein in A549 cells treated with BaP for 24 h was confirmed by Western blot (Fig. 5C). The ability of BaP to increase the expression of *CYP1B1 *is consistent with the general perception that this chemical can bind to the AhR, which is the transcriptional factor of *CYP1B1*. We have confirmed the presence of the AhR protein in this cell line although its expression does not appear to have been affected by the drug treatments (data not shown). We also examined the expression of other CYP genes (group 6 in table 1) and found no appreciable response to the treatments at any of the time points (data not shown).

## Discussion

To gain insights into the mechanisms through which chemicals interfere with cell cycle progression, we monitored cell cycle changes by flow cytometry analysis combined with cDNA microarray assay using cells from the same experiments. Cells treated with DMSO for 6 h showed well-defined G1 and G2/M phase peaks and a good proportion of cells distributed in S phase, indicative of proliferation status. The G2/M phase peak was smaller at 24 h and not readily discernable at 48 h and 72 h, indicating that cell growth had reached stationary phase at this late stage of the culture. An increase of cytoxicity was detected in cells treated with etoposide for 72 h. We examined the nature of etoposide-induced cytotoxicity and found no degradation of PARP and caspase-3, well documented features of apoptotic cell death (data not shown).

Although exposure to etoposide appeared to induce cell cycle arrest predominantly at G2/M phase, global gene expression analysis suggested that at the dose used (10 μM) the chemical has the ability to inhibit cell cycle progression at multiple stages. It effectively caused an accumulation of p53 protein, which leads to the up-regulation of its transcriptional gene targets. Included in these gene targets were *Waf1 *and *TGF-β*, both protein products are involved in the G1 checkpoints with *WAF1 *also being involved in G2/M checkpoint. Additionally, etoposide inhibited the expression of many other genes that function in the execution of cell cycle progression through S phase, G2/M transition and mitosis.

Although exposure to CLP induced a cell cycle profile similar to that induced by etoposide at the 24 h, there was a preferential inhibition of the expression of genes involved in G2/M transition. This contrasts with etoposide, which was more effective in inhibiting the expression of genes controlling both G2/M phase and mitosis.

BaP and PhIP treated cells showed no obvious phase specific effect on cell cycle progression over the period of study (72 h). The lack of effect of polycyclic aromatic hydrocarbon carcinogens on cell cycle in cells, including A549 cells, has been reported by others and described as stealth carcinogenesis [29]. Exposure to BaP and PhIP had little, if any, effect on the expression of cell cycle regulatory genes at the 6 h and 24 h time points. However, after prolonged treatment (48 – 72 h), BaP and PhIP induced alterations in the expression of many cell cycle regulatory genes. The effects exerted by BaP and PhIP on gene expression were not associated with dramatic changes in cell cycle profile, which may be due to a delay in the ability of the chemicals to influence transcription by which time the cells had achieved confluence.

p53 protein accumulation often occurs as an indicator of DNA damage [30,31]. The products of p53 transcriptional target genes are known to play important roles in multiple cellular biological processes, including cell cycle checkpoint control [32,33], DNA synthesis [34], DNA damage repair [26] and apoptosis [35]. P53 selectively regulates the expression of its targets in response to certain treatments [36]. Moreover, it has been found that some p53 target genes are also regulated in a p53-independent manner [37,38]. Our experiments in A549 cells showed that etoposide and CLP were almost equally effective in inducing the accumulation of p53 protein at 6 h of treatment, whilst BaP and PhIP failed to do so up to 72 h. We selected a number of p53 target genes to determine whether the dynamics of their expression was altered by the various chemical treatments applied. It was observed that both etoposide and CLP showed similar patterns of effects on the expression of some p53 target genes, including *Waf1, MDM2 *and *TGF-β *and *Bax*, although the etoposide effects were more prominent. The ability of etoposide and CLP to affect the expression of the p53 target genes was not reflected in changes in the expression of these protein products. This was exemplified by the observation that CLP was more effective than etoposide in upregulating the expression of MDM2 protein despite being less effective in upregulating the expression of *MDM2 *at mRNA level. These results suggest that there must be differences in the post-transcriptional regulation of the expression of MDM2 protein in response to the treatments of etoposide and CLP.

MDM2 acts as an E3 ubiquitin ligase which mediates autoubiquitination and ubiquitination of other proteins including p53 [39]. The balance between auto- and substrate-ubiquitination of MDM2 is modulated physiologically by posttranslational modifications, including sumoylation and phosphorylation. If SUMO conjugates to MDM2, its E3 ligase activity is shifted toward p53, while self-ubiquitination is minimized [40]. The tumour suppressor P19^ARF ^associates with MDM2 to inhibit the ubiquitination, export and subsequent degradation of p53 [41,42]. Given that MDM2 sumoylation is also stimulated significantly by ARF [43], SUMO and ubiquitin modifications appear to be mutually antagonistic. The switch in modification status is stress-responsive, because UV irradiation leads to loss of MDM2 sumoylation [44]. Further study will be needed to investigate whether CLP and etoposide induce different modification of MDM2.

More interestingly, it was observed that p53 protein was much reduced in cells treated with etoposide for 48 h and 72 h, compared with those treated with CLP at the same time points. This suggests that etoposide and CLP influence the expression of p53 by different mechanisms. The fact that etoposide remained effective in altering the expression of the so-called p53 target genes in the absence of p53 protein accumulation suggests that exposure to etoposide may also result in regulation of genes *via *p53-independent mechanisms. BaP and PhIP, neither of which effected p53 protein, selectively increased the expression of *Bax *and its protein product at 24 h but had little effect on other p53 regulated genes. Again it seems that exposure to BaP and PhIP can result in regulation of the expression of BAX *via *a p53-independent mechanism. The p53-independent regulation of BAX has been previously reported [38].

Among the many CYPs, CYP1A1 and CYP1B1 are major enzymes involved in the metabolism of procarcinogen PAHs to their DNA reactive species [45,46]. It has been shown that A549 cells express AhR and both CYP1A1 and CYP1B1 [25] and are able to activate BaP to form DNA adducts [[47] and our unpublished data]. In the present study we found that A549 cells treated with DMSO constitutively express more *CYP1B1 *mRNA than *CYP1A1 *mRNA (data not shown) The expression of *CYP1B1 *increased dramatically after treatment with DMSO for 48 h and 72 h compared with those treated for shorter times yet DMSO is not thought to induce this enzyme. It has been reported that *CYP1B1 *is a senescence related gene in human cells and in mouse cells [48,49], thus the late increase in the expression of *CYP1B1 *in DMSO treated cells may be related to confluence-mediated cell growth inhibition. BaP drastically increased the expression of *CYP1B1 *mRNA at early times with little effect on *CYP1A1 *mRNA (data not shown), suggesting that in A549 cells CYP1B1 may be the key enzyme in metabolizing BaP. The early upregulation of *CYP1B1 *in response to exposure to BaP was followed by downregulation of many cell cycle regulatory genes, supporting the proposal that *CYP1B1 *protein may have an important role in the regulation of cell cycle. Alternatively, the downregulation of key genes for cell cycle progression induced by BaP may be mediated by the interaction of AhR with other transcription factors.

By 72 h, the expression of key cell cycle regulatory genes in DMSO treated cells was lowest (measured at mRNA and protein level), indicating the status of growth inhibition. However many genes, particularly those involved in regulating the cell cycle in mitosis phase and in initiating DNA synthesis, were expressed higher in PhIP treated cells at this late time point, implying that cells treated with PhIP retain the potential to proliferate at confluence phase. At present it is unclear whether the effects of PhIP on cell cycle progression are due to DNA reactivity or to an epigenetic mechanism. However, our results are coincident with the report that PhIP activates the MAP kinase pathways in MCF-10A cells [50] and MCF7 cells [51]. The ability of PhIP to enhance cell growth signals under constraint culture conditions may be important to its carcinogenic properties.

## Conclusion

Studying mRNA expression in concert with cellular dynamics provides an effective way of understanding molecular mechanisms of action of chemicals that interfer with cell cycle progression. We have shown that exposure to the DNA intercalating chemicals etoposide and CLP can rapidly induce cell cycle disturbance by inhibiting the expression of multiple cell cycle regulatory genes, whereas the chemical carcinogens BaP and PhIP are insidious and pronounced cellular change requires more prolonged treatment. We conclude that the present work has identified a panel of genes that are responsive to the chosen chemicals and provides evidence supporting the proposal that genomic manipulation with chemicals will influence cellular outcome and that the nature and temporal aspects of the genomic response is predictive of prognosis.

## Materials and methods

All reagents were purchased from Sigma-Aldrich (Poole, UK) unless otherwise indicated.

### Cell culture and treatments

The human lung adenocarcinoma cell line A549 (European Collection of Cell Cultures, Wiltshire, UK) was cultured in Ham's F12 medium supplemented with l-glutamine (2 mM), 10% fetal bovine serum and 10 μg/ml gentamicin (all from Invitrogen, Paisley, UK) in a humidified incubator at 37°C with 5% CO_2_. When 70 % confluence was reached, cells were treated with DMSO (< 0.1% v/v), etoposide (10 μM), benzo(a)pyrene (25 μM), cryptolepine (2.5 μM) (gift from Dr Addae-Kyeremeh, University of Science and Technology, Kumasi, Ghana) and PhIP (50 μM) (purchased from Toronto Research Chemicals, Canada), for up to 72 h. All cells were harvested using 0.1% trypsin solution in EDTA.

### Flow cytometry

Cellular DNA content was determined by propidium iodide staining flow cytometry as described previously [52].

### Western blotting

Cell lysates (10 μg protein) were resolved by SDS-polyacrylamide gel electrophoresis, electroblotted onto nitrocellulose (0.45 μm), and blocked by incubation in 5% nonfat dry milk in phosphate buffered saline for 1 h at room temperature. The nitrocellulose was incubated with primary antibody overnight at 4°C, followed by secondary antibody conjugated to horseradish peroxidase for 1 h at room temperature. Detection was achieved using ECL kit (Amersham Life Science, UK). The antibodies against p53 (sc-6243), Bax (sc-493), cyclin A (sc-751), cyclin B (sc-752) and MCM7 (sc-9966) were purchased from Santa cruz, (California, USA). Antibody against p21/waf1 (CP74) was purchased from Neomarkers (Fremont, USA). Antibody against MDM2 (OP114) was purchased from CN Biosciences (Nottingham, UK).

### DNA Microarray Assay

Total RNAs were prepared with the RNeasy Mini Kit (Qiagen), according to manufacture's instruction. Ten μg of total RNA was reverse transcribed to cDNA by using T7-(dT)24 primer and Super Script Double-stranded cDNA Synthesis Kit (Invitrogen). Biotin-labeled cRNA was synthesized from cDNA by using ENZO bioArray High Yield RNA Transcript Labeling Kit (Enzo Diognostics). The cRNA samples were hybridized to human GeneChip arrays containing approximately 12,000 human genes (Human Genome U95A, Affymetrix). All analyses were performed at the Affymetrix core facility (Microarray Centre, Hammersmith Hospital, Imperial College London), in accordance with the MAIME protocol [53]. The average intensity of all genes on each chip was adjusted to 1,500 to allow for comparison and subsequent analysis [54].

### Gene grouping

All genes were categorized according to the MAPP files from the GenMAPP database [55]. Due to a failure in sample processing, data at 48 h was not available for agent CLP.

### Real-Time quantitative RT-PCR

Total RNA samples from preparations used for the GeneChip hybridisation were also used as templates for Real-time quantitive RT-PCR to confirm the microarray expression data. Reverse transcription was performed using SuperScriptII (Invitrogen). For each RNA sample, three cDNAs and one negative control (no transcriptase) were synthesized by using 2 μg of RNA sample and 0.2 μg/μl random hexamer primer, in total volume of 20 μl. RT-PCR was performed using Applied Biosystems 7900 sequencer. Primers and probes were purchased from Applied Biosystems and designed within an exon for each gene using the Primer Express program, version 1.5 (sequences are available on request). Each reaction contained 200 nM forward and reverse primers, 200 nM probe, 5 μl cDNA template, 12.5 μl Taqman mastermix 2× (Applied Biosystems) and ddH_2_O q.f to 25 μl. The cycling parameters were an initial 50°C for 2 mins and 95°C 10 mins, followed by 40 cycles of 95°C for 15 s and 60°C for 1 min. We used primers and a probe corresponding to the housekeeper gene 18 s rRNA to which we normalized the expression of genes of interest. Data was retrieved from SDS2.0 software (Applied Biosystems) and analysed using the SAS system for Windows: Release 8.01 TS level 01M0.

### Validation of the DNA microarray data

We employed real-time PCR to quantify 9 DNA-repair gene targets (Table 2) and used the data to validate the DNA microarray assay. Results of the two assays were compared and the correlation coefficients are presented in table 2. The two assays showed good consistency with an overall correlation of > 0.73.

**Table 2:** Correlation between DNA repair gene expression determined by cDNA microarray assay and RT-PCR.

**Treatment**	**Correlation Coefficient**	
	**6 h**	**24 h**
DMSO	0.75	0.70
ETOP	0.76	0.80
BaP	0.69	0.79
CLP	0.62	
PHIP	0.71	0.77

The expression of 9 genes, including APEX (M80261), ATM (U26455), Pol-beta (D29013), c-fos (V01512), Gadd45 (M60974), RAP1 (M63488), TPA (M15518), XRCC1 (NM 00639) and XRCC2 (Y08837) were analyzed using real-time quantitative RT-PCR, and the results were compared with that derived from DNA microarray assay.

## Abbreviations

BaP, benzo(a)pyrene; DMSO, dimethyl sulphoxide; PAH, polycyclic aromatic hydrocarbon; PhIP, 2-amino-1-methyl-6-phenylimidazo [4,5-b]pyridine; CLP, Cryptolepine; ETOP, etoposide.
